# Exploring the role of learning through play in promoting multimodal learning among children: a pilot study in Chinese first-tier cities

**DOI:** 10.3389/fpsyg.2023.1103311

**Published:** 2023-05-30

**Authors:** Xiuhan Li, Xiaoman Zhang, Yongle Zhao, Lu Zhang, Junjie Shang

**Affiliations:** ^1^Faculty of Artificial Intelligence in Education, Central China Normal University, Wuhan, Hubei, China; ^2^Honghe University, Mengzi, Yunnan, China; ^3^Beijing University of Posts and Telecommunications, Beijing, China; ^4^Graduate School of Education, Peking University, Beijing, China

**Keywords:** learning through play, playful learning, student-centered learning, multimodal learning pedagogy, multimodal learning ecosystem

## Abstract

Play is an effective approach to engaging children in learning as an alternative to traditional lecturing. The Learning through Play (LtP) approach involves various modes of learning participation, including multi-sensory participation, interpersonal interaction, and hands-on operation, which can effectively motivate children to learn. This study implemented an LtP pilot survey in several first-tier cities of China, which included questionnaires and interviews. The results present the basic ecology of LtP in China, LtP effects on children's multimodal learning. We found that LtP has gained great popularity, both conceptually and practically, in China. LtP stakeholders recognize the behavioral, cognitive, and affective effectiveness of LtP for children's learning. The factors influencing the effectiveness of LtP include its structural weaknesses, the subjects involved, the environment, and culture. This study provides a reference for promoting the theory and practice of children's multimodal learning with a playful approach.

## 1. Introduction

Play is appealing for children, making it an important factor in their learning process. Education scholars, from Confucius and Socrates to the present day, have agreed that play is inextricably linked to children's learning. Play has an important role in promoting children's physical, mental, cognitive, social, and emotional development (Goldstein, 2012). Children are more likely to choose learning activities that they enjoy and to remember enjoyable learning experiences (Zosh et al., 2018). Student-centered learning has been emphasized in social and educational policies for many years. From creating learning environments to designing learning content and implementing learning activities, student-centered learning focuses on what students need. Many educators are aware of the importance of stimulating students' intrinsic motivation to enhance their learning performance. However, the learning experience and learners' physical and mental states are also important (Turdieva and Olimov, 2021). Learning through play (LtP) is a significant and effective approach to promoting children's all-around learning.

Metaari's Global Game-based Learning Market report (2018–2023) found that young children are the main consumers of educational and serious games (Adkins, 2018). China represents the largest video games market worldwide, with an estimated 619.5 million players, most of whom are children and young adults (China Games Market., 2018). Thus, LtP has great potential in both educational and economic terms in China. Globally, many new LtP spaces have recently emerged. They are aimed at a wider range of children than early-learning centers, and some even cater to middle school students (Hassinger-Das et al., 2020). LtP spaces share the characteristics of playful learning in terms of venues, devices, resources, and activities. Examples of such spaces include Atelier Caracas, Lego Education Learning Centers, and SolBe Supernormal (De Arce, 2018). In playful learning environments and through playful learning activities, learners engage in multimodal information exchange by interacting with events, people, and tools (Papagiannakis et al., 2013). The openness, diversity, and enjoyment experienced through LtP suggest that a multimodal learning mechanism is involved. Recent information technology developments have helped provide children with access to multimodal worlds (Kopcha et al., 2021). Therefore, identifying current approaches to LtP and understanding how it can facilitate children's multimodal learning experiences may benefit them. In this study, we investigated how LtP can help facilitate children's multimodal learning through a pilot field study conducted in China.

### 1.1. LtP

Before examining LtP, the notion of play should be discussed. Play, which is often opposite to work, is a type of social constructionist behavior that is informal, fun, and active (Fromberg and Bergen, 2012). Play is an essential part of human culture and has evolved as a safe and fun way for young children (and some animals) to learn about themselves and their environments (Yogman et al., 2018). Play can also be a serious and high-stakes activity that involves compulsory tasks, e.g., group games (Brown, 2009). Froebel (1885) described play as encompassing all spheres of a child's life, as it not only attends to a child's physical needs to be active but also provides space for nurturing, exploration, joy, and contentment.

LtP has been regarded as an effective learning method, as it promotes children's development and is fun, active, engaging, meaningful, socially interactive, and iterative (Zosh et al., 2017). Fun is the essential feature of LtP in terms of learning. An important goal of education is to make people happy or advance their pursuit of happiness (Noddings, 2003). Through active involvement, learners think and act rather than simply watching and listening. If they are fully engaged, they may forget where they are and lose track of time (Whitebread et al., 2017). Effective LtP learning materials or activities can be meaningful for children at a narrative level (even meaningful fantasy). Problem-solving can be meaningful at a level consistent with learners' cognitive development, as they can gain knowledge and skills based on their efforts. In addition, through LtP, learners can effectively interact with others. Although children can engage in LtP activities alone, these activities are much more effective in social settings (Zosh et al., 2018). Iterative designs of playful activities, games, or toys can result in different experiences and outcomes depending on the situation or participants (Whitebread et al., 2017). This design feature involves the creativity and versatility of play activities, such as building blocks. Children may have different ideas for building methods each time they consider the individual blocks, and their experiences will then be improved.

Mardell et al. (2019) noted that any joyful activity can lead to LtP if a child is active and cognitively engaged. This can help them find meaning in what they do or learn. Iterative thinking (experimentation, hypothesis testing, and so on) and social interaction with peers and adults are also important. Some studies have referred to LtP as playful learning or gamified learning. In its Innovating Pedagogy Report of 2019, the UK's Open University suggested that LtP, or gamified learning, is one of the most significant learning approaches of the 21^st^ century. The report divided LtP into role-playing, mobile digital, educational, and gamification games. Zosh et al. (2017) distinguished between free-play, guided, and game-play LtP. The LtP approach can be implemented through free-play activities in designated public spaces, both outdoors and indoors, or in informal settings within residential communities. Children may be provided with resources and space to engage in free play. Guided-play LtP usually happens at school and in LtP centers, where teachers and practitioners are required to help and organize the children participating in the activities. Game-play LtP generally involves playing virtual educational games on digital devices. Several educational technology businesses, such as Lego, have developed innovative and smart learning centers (Liu et al., 2017).

### 1.2. Multimodal learning

Modes are channels of information that convey meaning; they include photos, illustrations, speech, writing, print, music, movement, gestures, facial expressions, and colors (Zhang et al., 2010). Yelland (2018) suggested that building an early learning ecosystem, or ecology, in the information era should focus on the multimodal, not the digital. Human cognition is the sum of multimodal learning (Ivanovic et al., 2018). We establish interactions with complex environments through our visual, auditory, and other sensory systems and construct meaning from this external information through the cerebral cortex (Di Mitri et al., 2022). Multimodal learning aims to enable children to process and associate information from multiple channels (Schneider et al., 2018). Some studies have suggested that multimodal learning involves multiple learning environments, behaviors, and cognitive processes (e.g., Blikstein and Worsley, 2016). The emergence of screen-based digital devices has enabled children to interact with multimodal (digital and printed) text as required (Rowan and Honan, 2005). Kalantzis and Cope (2012) suggested that in the 21^st^ century, multimodal learning ecosystems should be designed to provide learners with various social experiences in dynamic communities. People are capable of adapting to changing circumstances if they gain rich experiences in their current circumstances. Several studies on learning styles have considered different information processing channels as a form of multimodal learning, as they involve sight, hearing, and touch (Yelland, 2011). Fleming (1995) VARK model distinguishes four channels for humans' multimodal learning: visual, auditory, reading and writing, and kinesthetic.

The development of information technology has led to increasingly multimodal forms of learning for children in the 21^st^ century (Yelland, 2018). Children in the present times have access to abundant resources for both learning and entertainment, enabling them to effectively communicate their understanding of the world and derive meaning from it (Yelland, 2018). Through mobile devices, children these days have experience interacting with multimedia (visual, audio, and linguistic), along with social communities (computers, teachers, peers, and parents), which have been shown to be an effective combination (Yelland, 2018). Multimodality-based learning helps develop “21^st^ century skills” (Trilling and Fadel, 2009; Yelland, 2016), such as problem-solving, creativity, critical thinking, collaboration, and communication, which may not be fostered by traditional lectures (Gellevij et al., 2002; Bell, 2010). Students need to be provided with authentic learning contexts that not only involve processing learning information but are also a means of pleasurable self-expression and hands-on inquiry in various contexts (oral, aural, linguistic, visual, and kinesthetic). For example, an audio-visual teaching mode has been found to improve students' listening and speaking skills, their ability to use language, ability to learn independently, and attitudes toward learning language (Bagila et al., 2019).

In multimodal learning, data from various sources can be collected and integrated to support learning analysis. The research field of multimodal learning analytics (MMLA) combines multimodal data with learning feedback (Worsley et al., 2021). MMLA techniques provide a foundation for smart multimodal learning apps and support numerous innovative learning activities. Through real-time data integration, learners and teachers can recognize and precisely intervene in the learning process (Schneider et al., 2018). Eye movement data, electroencephalograms, measurements of event-related potential, expression recognition data, physiological data, and questionnaire data can be integrated with traditional learning data for analysis to create multi-modal integration analysis platforms (such as iMOTIONS), which provide a method for scientifically assessing students' learning performance (Yiew et al., 2023).

Although we found no studies that fully explained the relationship between LtP and multimodal learning, current empirical evidence suggests that LtP has the potential to offer students multimodal learning experiences. Students can use the sensory information provided in gamified environments and activities to establish interactions with social communities and complete game tasks (Hassinger-Das et al., 2018; Yogman et al., 2018). LtP and multimodal learning have a common underlying logic; therefore, designing multimodal learning activities and assignments should result in playful, creative, and/or engaging experiences. Several studies have explored the connections between LtP and multimodal learning. Li and Chu (2021) investigated children's engagement in Hong Kong primary schools in an LtP program called “*Reading Battle*.” They could read books, participate in “battles,” gain points, and write and “publish” their own storybooks within the Reading Battle LtP platform. This project has been implemented in more than 50 primary schools in Hong Kong since 2014. Children's reading achievement was assessed using multimodal data, such as the time spent using an LtP app, the accuracy of answers, points and leaderboard scores, and peer evaluation of the created storybooks. The results showed that the multimodal reading experience can help increase children's interest in reading and literacy. Other programs use multimodal books or gamified reading apps to develop children's literacy, particularly for those who struggle as readers (Rowe and Miller, 2016; Yelland, 2018). Yelland (2018) suggested that play enables children to build on and extend their “real-world” perceptions in dynamic, interactive, and multimodal contexts. In addition, 3D video experiences, a popular element of LtP environments, are viewed as a type of multimodal learning scaffold. They embody multimodal learning by offering theoretical knowledge, immersive 360-degree video experiences, and reflection in the same exercise (Haugan et al., 2023). Gee (2007) explained that multimodality is an important principle of 3D video games, as the playing context can provide multimodal data for analysis, such as gameplay, facial expressions of emotions, and eye gaze (Emerson et al., 2020). Thus, we proposed and constructed an LtP framework to build a multimodal learning ecology for children. We explored this framework and its contribution to multimodal learning. Our main research question is as follows: How does LtP support children's multimodal learning? This leads to three sub-research questions:

- How has LtP been applied in the urban areas of China?
- How does LtP contribute to children's multimodal learning experiences?
- What can affect the development of LtP?

## 2. Research methodology

**Figure 1:**
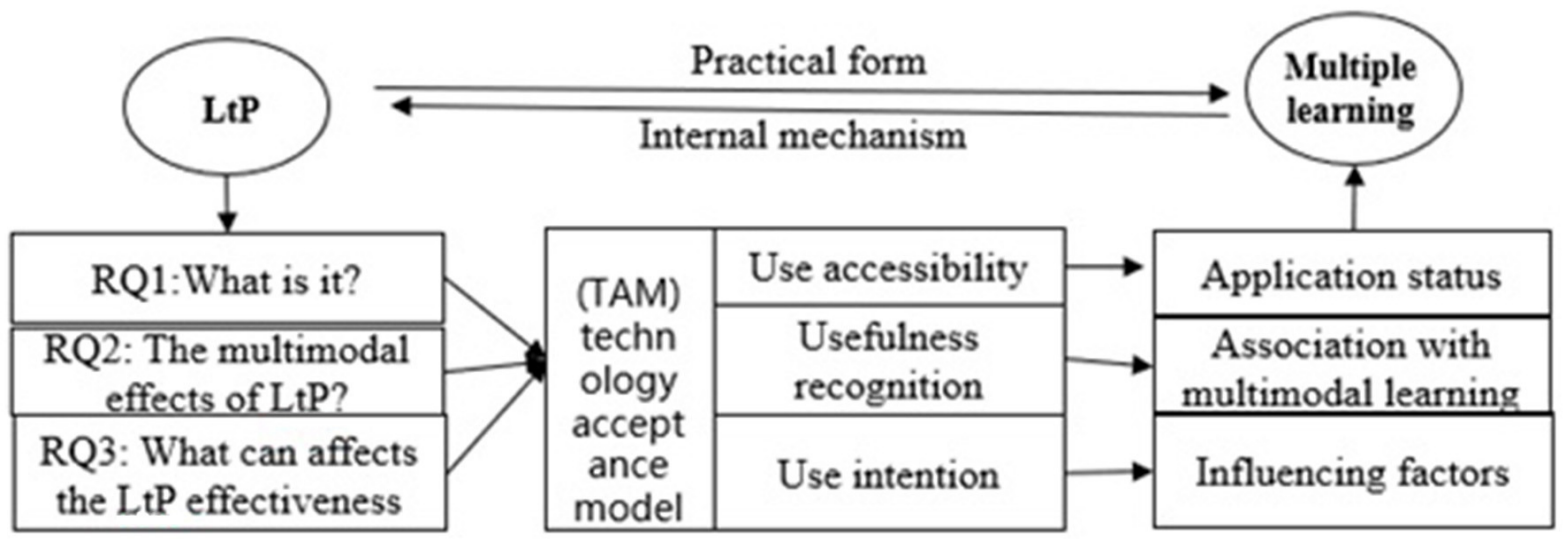
Research framework.

The goals of the present study were three-fold: (1) to describe the current status of LtP in China; (2) to identify the multimodal learning effects of LtP; and (3) to provide practical suggestions for incorporating playful learning. The research framework is shown in Figure 1. We regarded playful learning as a practical form of multimodal learning and assumed that multimodal learning can explain the internal working mechanism of playful learning. The technology acceptance model (TAM) formed the theoretical basis of our study. For RQ1, information on the technologies (spaces, devices, and activities) used for playful learning and stakeholders' attitudes were obtained through a questionnaire survey to illustrate how playful learning is applied in China. The effects of LtP were explained from the perspective of multimodal learning. Finally, suggestions for the LtP application were identified from stakeholders' interviews.

### 2.1. Research methods

We implemented a mixed-methods research procedure to explore LtP practices in China, involving questionnaires and interviews. RQ1 was addressed through a questionnaire survey, and RQ2 and RQ3 were addressed through interviews and open questions in the questionnaire. The quantitative approach of this research used a questionnaire to investigate the status of LtP in Chinese first-tier cities. A paper-and-pencil questionnaire was developed and implemented in the class. The qualitative part of the study was implemented through semi-structured interviews. The interviewees were sampled from four first-tier cities in China: Beijing, Shenzhen, Hangzhou, and Wuhan. Children and teachers from the abovementioned three schools were recruited and participated voluntarily. Reflexive thematic analysis (RTA) was used to assess the interview data. Codes in RTA represent the researchers' interpretations of meaning across the dataset. The effects of LtP were coded as behavioral, cognitive, or affective, following Sawyer (2005). The answers relevant to RQ3 were abstracted into two dimensions: weaknesses and influencing factors. Themes were identified based on the outcomes of data coding and iterative theme development. Several open questions were added to the questionnaire to enrich the qualitative data.

### 2.2. Research instruments

A questionnaire (see Appendix 1) was administered to measure the status of LtP in the sampled cities. In addition to questions on the respondents' sociodemographic characteristics (e.g., age, grade, academic performance, and gender), the questionnaire consisted of multiple-choice questions, questions based on Likert scales, and open questions. The students were asked to report their academic ranking rate in the last term as an indicator of the variable of academic performance. The multiple-choice questions covered the design of LtP spaces, technologies, devices, and activities. The Likert scale questions included two sub-scales: acceptance of LtP (15 items, Cronbach's α = 0.775) and evaluation of LtP effectiveness (12 items, Cronbach's α = 0.857). Cronbach's α values were used to assess the reliability of the scale terms, the threshold of which should be >0.70. The sub-scale measuring the acceptance of LtP consisted of three dimensions based on the TAM. The other sub-scale assessed the respondents' evaluation of LtP multimodal effects in behavioral, cognitive, and affective dimensions. More details regarding the structure and reliability of the subscales can be found in Table 1. Moreover, the parents' attitudes toward LtP were rated by the children from “much supported” to “no support.” The Likert scale items were adapted from the literature to match the context of this study. The respondents were asked to indicate the extent to which they agreed with each statement on a 5-point Likert scale (from 1 = “strongly disagree” to 5 = “strongly agree”).

**Table 1:** The structure and reliability of the Likert subscales.

**Subscale**	**Dimensions**	**No. of items**	**Cronbach's α**
Acceptance of LtP	Use accessibility	5	0.637
	Usefulness recognition	5	0.882
	Use intention	5	0.734
Evaluation of LtP effects	Behavioral	4	0.656
	Cognitive	4	0.809
	Affective	4	0.626

The three open questions were designed to elicit non-preset answers. We asked the participants to indicate the LtP spaces they liked the most and explain why, report the difficulties and challenges they had encountered with LtP, and provide suggestions for improving LtP approaches. After the initial questionnaire was developed, two professionals and three primary students conducted an iterative manual review process to check its completeness, appropriateness, and wording. We revised and refined the questionnaire according to their feedback. For example, some Likert scale items were eliminated or modified because their meaning was ambiguous, thus keeping the children's workload to a minimum and enhancing the psychometric properties of the instrument.

The interview question design followed the research framework in Figure 1. Stakeholders in LtP (i.e., children, teachers, parents, and educational administrators) were invited to share their perceptions of the effects of LtP, factors influencing LtP, challenges to LtP, and suggestions for improving LtP. The outline of the semi-structured interviews is provided in Appendix 2. The interview data were coded by two independent coders based on the abovementioned framework. The inter-rater reliability of the coding was measured by Cohen's kappa coefficient (the percentage of agreement between coders) in NVivo 10. The two coders engaged in iterative discussion until the kappa coefficient of each node was above 0.75, which is considered to show an excellent level of agreement (Fleiss et al., 2013).

### 2.3. Sampling

The survey sample consisted of 1,495 children from nine primary schools in Beijing and Shenzhen, which are typical first-tier cities in the north and south of China, respectively. The primary schools were selected through a non-probability snowball sampling method. The research team first contacted three schools that had cooperative relationships with Peking University (the principal investigator's institution) and encouraged them to introduce six other schools. The two selection criteria were that the schools were public and were not in suburban areas. The 1,495 sampled students were aged between 10 and 12 and were in the fourth and fifth grades. The sample was 48% women. For the interviews, we invited four types of LtP stakeholders via the Internet. Thirty LtP stakeholders, including 11 children, eight parents, eight teachers, and four educational administrators, enrolled in the interviews voluntarily. More information about the interviewees is shown in Table 2.

**Table 2:** Interviewees' information.

**Information**		**Children**	**Parents**	**Teachers**	**Education administrators**
Gender	Women	5	8	5	1
	Men	6	0	3	3
City	Beijing	2	3	4	2
	Shenzhen	2	2	2	0
	Wuhan	6	2	1	1
	Shanghai	1	1	1	1
Notes		Preschool-1 third grade-2, 4^th^ grade-5, 5^th^ grade-2, 6^th^ grade-1.	Mother-8	30–45 years old	Principal-2, Policymaker-2

## 3. Results

### 3.1. RQ1: the status of LtP in Chinese first-tier cities

Before assessing the children's LtP experiences, we first examined their acceptance of LtP in three dimensions, each measured on a 5-point Likert scale. The results are shown in Table 3. The overall acceptance score was 4.19, showing that the students had a positive attitude toward and were receptive to LtP. Among the three dimensions of LtP acceptance, “usefulness recognition” gained the highest score (M = 4.33, SD = 0.82). We conducted a *t*-test to identify potential gender differences in the students' acceptance of LtP, and the results showed that girls (M = 4.24, SD = 0.60) scored significantly higher than boys (M = 4.15, SD = 0.73), with a *p*-value of 0.031 (< 0.05).

**Table 3:** Students' acceptance of LtP.

**Dimension**	**Mean**	**SD**
Use accessibility	4.14	0.68
Usefulness recognition	4.33	0.82
Use intention	4.00	0.88

1, totally disagree; 5, totally agree.

We identified five main types of LtP spaces: school spaces, community spaces, public science and technology museums, outdoor-themed playgrounds, and commercial play centers. The questionnaire results indicated that the children regarded these LtP spaces positively (see Table 3). Outdoor-themed playgrounds, such as theme parks and nature parks, were the most liked LtP spaces (M = 2.43; SD = 1.4). The children's evaluations of commercial play centers (e.g., career experience centers and community spaces) were similar. School LtP spaces, such as STEM rooms and spaces in or around communities, followed. Boys and girls differed significantly in their preferences regarding LtP spaces. The results indicate that boys preferred commercial play centers and public science and technology museums, while girls preferred outdoor-themed playgrounds and school-based LtP spaces.

The questionnaire data showed that the children's favorite LtP environments were commercial edutainment centers with extensive resources and access to high-tech devices. Blended learning spaces involving online and offline experiences stood out. As **Figure 6** shows, new LtP centers typically have striking, playful learning characteristics regarding decoration, equipment, and interactive activities.

**Figure 2:**
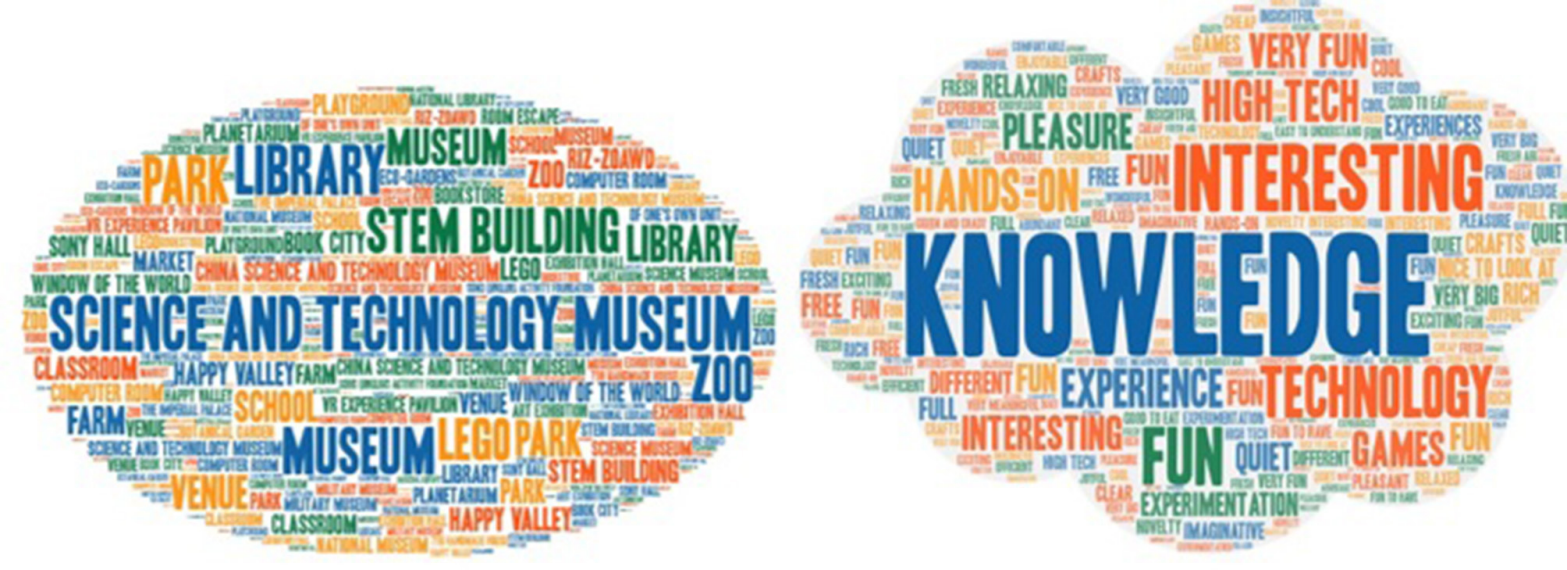
LtP places recommended by the students and their corresponding reasons.

We also designed an open question to invite the children to provide examples of LtP spaces that they often visited and explain why. We extracted keywords from the resulting data and imported them into an online micro-word cloud platform (https://www.weiciyun.com/) to generate a word-cloud map, as shown in Figure 2. We found that, although the children liked outdoor spaces the most, they frequently went to public indoor venues, such as science and technology halls, museums, and libraries. When they explained why, “knowledge” was found to be their main consideration, followed by “fun.”

**Figure 3:**
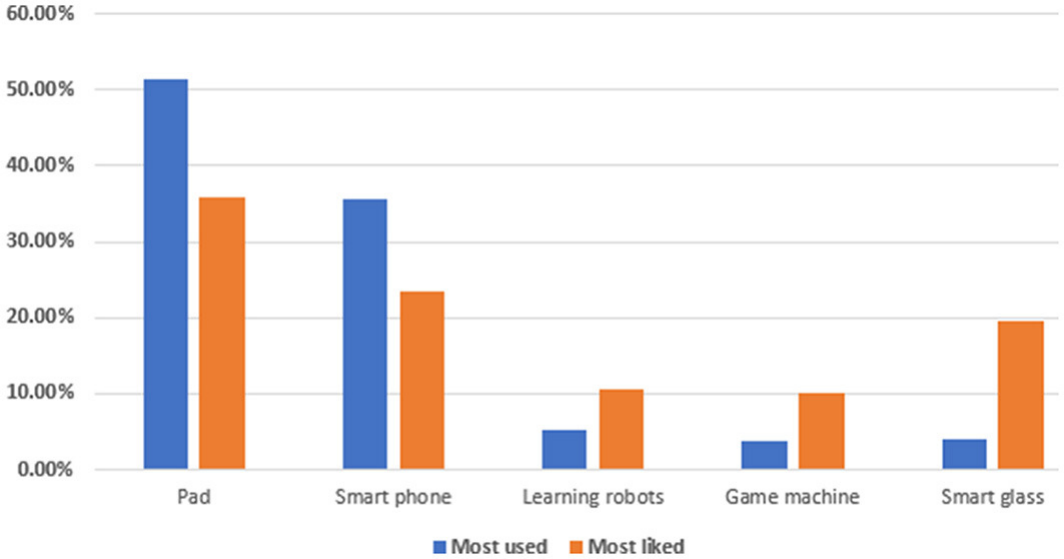
Children's devices and tools for LtP.

Regarding the devices and tools used, nearly all children reported that they could access at least one device for LtP. These devices and tools are shown in Figure 3. The tablet was the most used and most liked device, followed by the smartphone. Although learning robots and smart body equipment (e.g., smart glasses and gamepads) are increasingly common, they are not yet popular with families and thus will not be useful for children's LtP for some time.

Table 4 shows the children's level of enjoyment of LtP activities in the spaces. “Watching videos and experiencing V.R.” was the most popular activity, closely followed by “participating in LtP group projects.” The traditional learning activities of reading, writing, and viewing an exhibition were significantly less popular than the first two types of activities. It seems that the children preferred activities with more multimedia involvement and more participation modes. The results of a *t*-test indicated that boys preferred high-tech LTP activities, while girls preferred LtP project activities with more communication and collaboration.

**Table 4:** The degree to which children liked LtP activities.

**Type**	**Mean**				***t*** **-test**	
	**Overall**	**SD**	**Girls**	**Boys**	***t***	***p*** **-value**
Watching a video and experiencing VR	2.12	1.38	2.24	2.00	−3.368	0.01
Participating in LtP group projects	2.15	1.39	2.04	2.25	2.987	0.03
Viewing an exhibition	2.91	1.49	2.79	3.05	−3.332	0.01
Reading and writing	2.92	1.54	2.83	3.00	2.053	0.04

1, liked most; 5, liked least.

**Figure 4:**
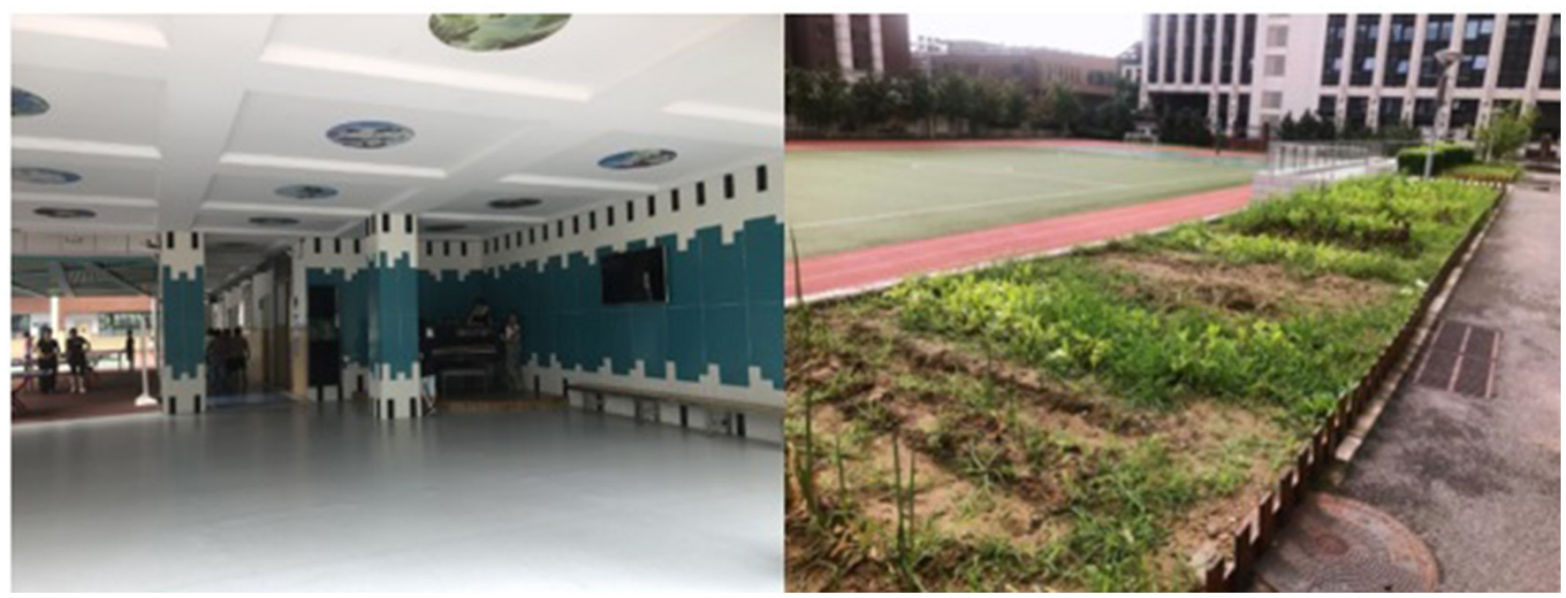
Piano Hall and students' self-managed farm at schools.

Multimodal learning involves various environments, resources, and tools. Schools providing LtP are personalized and fun venues that do not merely offer a production-line approach to learning. The interviewed teachers reported that their schools had many multi-functional classrooms to encourage children's LtP, which involved calligraphy, handicrafts, STEM, cartoons, dancing, and robots. Student A described his school, the Beijing Longyue Experimental Middle School, as a “gamified fairyland.” At this school, children could build “future towns” with farms, bazaars, tokens, and other gamified elements. Students could even plant crops on farms. At another primary school in Wuhan, the school hall was decorated in a “piano” style and had a piano in it that students could play when they wanted to. Figure 4 shows the Piano Hall and a farm self-managed by students in a “future town.”

**Figure 5:**
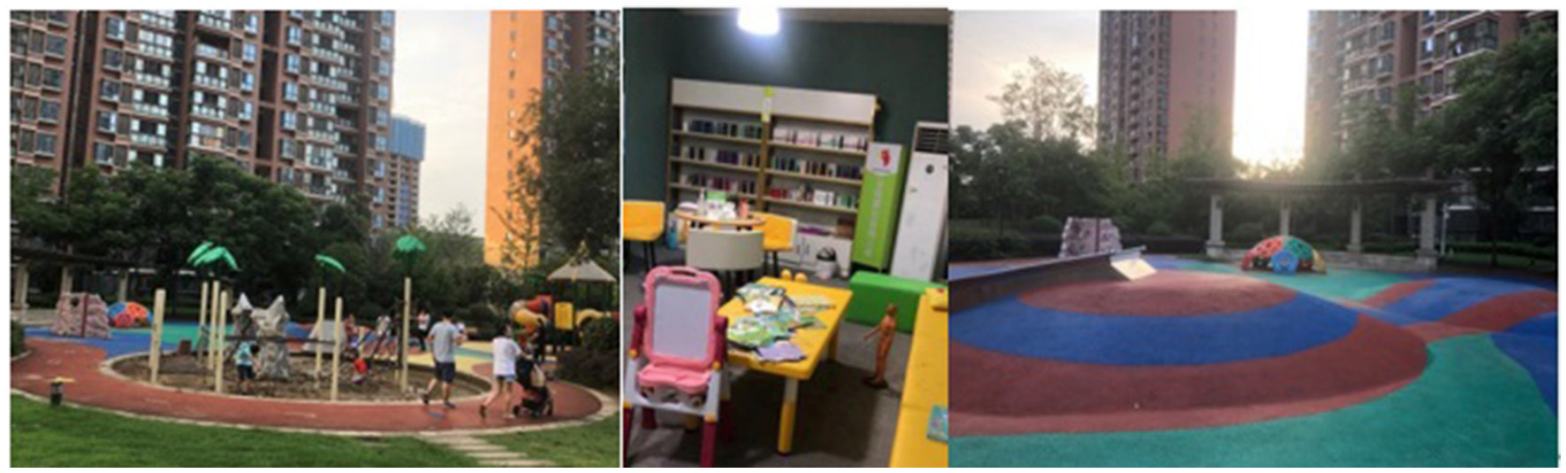
Examples of LtP spaces in living communities (retrieved from this fieldwork).

**Figure 6:**
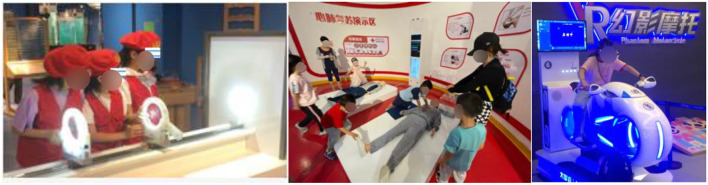
Examples of LtP spaces in commercial edutainment centers (retrieved from this fieldwork).

Schools are in danger of becoming irrelevant if they do not offer the experiences and materials that are available to children in their daily lives. LtP provides children with various learning environments beyond the classroom. Several living communities are currently providing LtP spaces for children, as learning and personal development are extremely important to families. The images in Figure 5 were provided by a parent, showing the LtP spaces in her living community. She said that these spaces included several activity rooms where children could read, paint, watch TV, and play with neighbors. The children's favorite LtP environments were commercial edutainment centers with extensive resources and high-tech devices, as shown in Figure 6.

As Table 5 shows, the children's top three requirements for LtP were that it should be hands-on, high-tech, and group-based. First, the children liked LtP activities in which they could become deeply involved. Second, they preferred to participate in high-tech LtP activities using smart devices. Third, they liked collaborative LtP projects more than solo projects. Fourth, they appreciated LtP organizations that support parent–child LtP activities. Fifth, they preferred open outdoor LtP spaces more than indoor spaces. Sixth, they liked colorful and comfortable LtP spaces. Finally, they preferred to have tutors and teachers guide and help them when participating in LtP. Girls and boys differed significantly in their preference for “high-tech devices and tools” and “colorful and comfortable spaces.” This result was consistent with the finding (reported in Table 6) that boys liked the information technology elements of LtP more than girls did.

**Table 5:** Students' LtP requirements.

**Type**	**Mean**				***t*** **-test**	
	**Overall**	**SD**	**Girls**	**Boys**	***T***	***p*** **-value**
Hands-on activities	4.50	0.93	4.52	4.49	−0.64	0.52
High-tech devices and tools	4.33	1.03	4.25	4.20	4.566	0.00
Group LtP activities	4.36	1.06	4.38	4.34	−0.65	0.51
Together with parents	4.31	1.09	4.34	4.28	−1.18	0.24
Open and outdoor LtP spaces	4.27	1.06	4.25	4.28	0.47	0.63
Colorful and comfortable spaces	3.82	1.27	3.93	3.71	−3.32	0.01
With tutors or instructors	3.73	1.30	3.71	3.74	−0.33	0.75

5, very important; 1, not important.

**Table 6:** The popularity of LtP spaces with the children.

**Type**	**Mean**				***t*** **-test**	
	**Overall**	**SD**	**Girls**	**Boys**	***t***	***p*** **-value**
Outdoor-themed playgrounds	2.43	1.40	2.20	2.64	6.124	0.00
Commercial play centers	2.69	1.50	3.05	2.34	−8.803	0.00
Public science and technology museums	2.69	1.59	2.94	2.46	−6.235	0.00
School spaces	2.75	1.49	2.62	2.87	3.260	0.01
Living community spaces	2.87	1.48	2.72	3.00	3.615	0.00

1, liked most; 5, liked least.

The results of the questionnaire indicated that the variables of parents' roles, educational background, and family socioeconomic status (SES) significantly influenced parents' input into their children's LtP. The respondents indicated that the time spent playing with their fathers was significantly longer than that with their mothers. Parents with higher levels of education and a higher SES spent more time on their children's LtP.

### 3.2. RQ2: how does LtP contribute to children's multimodal learning experiences?

We analyzed the multimodal learning effects of LtP following Sawyer (2005) three-dimensions of learning: behavioral, cognitive, and affective. We explored the degree to which students agree that LtP can help improve multiple learning behaviors, cognitive competencies, and learning emotions. The data were drawn mainly from the questionnaires and the interviews with the children, parents, and teachers. First, we investigated the children's perceptions of the effectiveness of LtP on multimodal learning experiences at three levels. Details of the scale items can be found in Appendix 1. As shown in Table 7, the children strongly agreed that LtP has multimodal effects at the behavioral, cognitive, and affective levels. The highest score was for the affective level. The item with which the students most strongly agreed was “I find that LtP can make my learning more interesting and engaging,” with a score of 4.54. We used a *t*-test and Pearson's test to conduct a correlation analysis between the children's scores and two dependent variables (gender and academic performance). Girls (M = 4.49) scored higher than boys (M = 4.41) for the affective dimension.

**Table 7:** Children's evaluation of the multimodal effects of LtP.

**Dimension**	**Mean**	**SD**	***t*** **-test**	
			**Gender**	
			***t***	***p*** **-value**
Behavioral	4.22	0.73	−1.48	0.14
Cognitive	4.39	0.75	−1.96	0.05
Affective	4.45	0.78	−2.13	0.03

1, strongest disagreement; 5, strongest agreement.

We used the regression analysis of the Structural Equation Model (SEM) in AMOS software to explore the relationship among children's accessibility of LtP (CAL), children's academic performance (CAP), parents' attitude toward LtP (PAL), and children's perceived multimodal effects of LtP (CPMEL). The parameter estimate results in SEM are shown in Table 8. The predictive paths from CAL and PAL to CPMEL are significant (*p* < 0.01), while those from CAP to CPMEL are not significant (*p* > 0.3). The final regression model's fit was satisfactory (NFI=0.73; average path coefficient of CAL = 0.881).

**Table 8:** Parameter estimates in SEM (*n* = 1,495).

	**Estimate**	**S.E**.	**C.R**.	**PLabel**
CPMEL-Behavioral ← Academic_performance (CAP)	0.014	0.016	0.863	0.388
CPMEL-Affective ← Academic_performance (CAP)	0.017	0.017	1.005	0.315
CPMEL-Cognitive ← Academic_performance (CAP)	0.000	0.016	0.001	0.999
CPMEL-Behavioral ← Use_accessibility (CAL)	0.822	0.030	27.545	^***^
CPMEL-Cognitive ← Use_accessibility (CAL)	0.895	0.030	29.819	^***^
CPMEL-Affective ← Use_accessibility (CAL)	0.926	0.031	30.068	^***^
CPMEL-Behavioral ← Parent_attitude (PAL)	−0.146	0.019	−7.678	^***^
CPMEL-Cognitive ← Parent_attitude (PAL)	−0.175	0.019	−9.155	^***^
CPMEL-Affective ← Parent_attitude (PAL)	−0.182	0.020	−9.232	^***^

CPMEL, children's perceived multimodal effects of LtP; CAP, children's academic performance; CAL, children's accessibility of LtP; PAL, parents' attitude toward LtP.

^***^p < 0.01.

**Figure 7:**
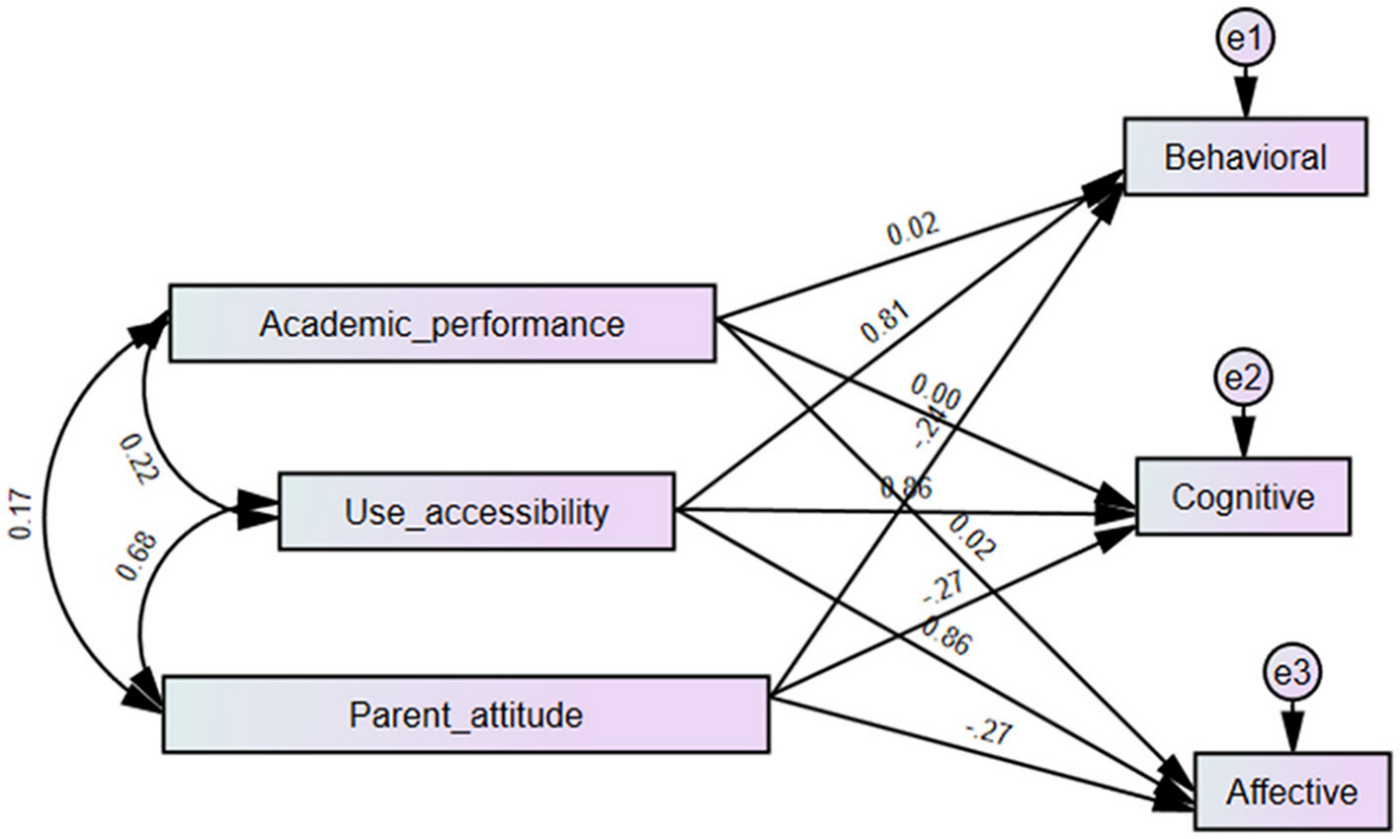
The final model predicting students' perceived LtP effects.

As shown in Figure 7, the predictive relationship between CAP and LtP effects is not significant, meaning that not only top-ranking students have high recognition for LtP multimodal effects. CAL has a positive predictive relationship (β = 0.881; *p* < 0.01) with CPMEL. It was easy to understand that only when students use LtP frequently can they gain more benefits from it. It was aslo found that PAL has a small negative predictive relationship (β = −0.26; *p* < 0.01) with CPMEL. This conclusion might be due to the parent–child relationship. Children with a worse relationship with their parents usually like LtP more than those with a better relationship.

We coded the interview materials based on Sawyer (2005) framework of LtP's effects on learning experiences. We identified 46 nodes (subthemes) at the behavioral, cognitive, and affective levels (see Table 9).

**Table 9:** Coding results for the learning effects of LtP.

**Theme**	**Nodes (sub-themes)**	**Cited examples**
Behavioral (15)	Game, activities, hands-on, tools, props, learning habits	*I found that my child has become braver in LtP activities and expressing himself. —Parent A* *I persist in using an online gamified English learning platform to gain points. —Child A* *I often play with Legos and lose track of time. —Child B*
Cognitive (11)	Knowledge, memorable, sense of ceremony, deep understanding, horizons	*My child likes to go to various kinds of museums. He has gained much knowledge. —Parent B* *I like competitions, props, rewards, badges, and leaderboards as they can create a sense of ceremony in learning. —Child C*
Affective (20)	Interesting, learning motivation, engaged, relationship, personality, decompression	*Children are very interested in game-based learning, which holds their attention. —Teacher A* *Children can suffer from great stress, and LtP can help them avoid anxiety. —Parent C* *I like teacher Li because she sets funny games in the class. Her class is very interesting. —Child D*

#### 3.2.1. Theme 1: LtP can trigger multimodal learning behaviors

The interviewees believed that LtP could promote various learning behaviors, as it is interesting and engaging. Traditional learning behaviors include listening, reading, writing, and speaking, while LtP involves multimodal learning activities and leads to different types of behavior. LtP pedagogy is an effective method of systematically connecting and integrating specific learning behaviors through speech, gesture, and physical movement. Hands-on, project-based, game-based, problem-solving, inquiry, and collaborative learning approaches can be combined. Games and gamification are core mechanisms of LtP activities, as they can motivate children to perform expected behaviors. With the support of teaching props and technologies such as cards, iPads, dice, hats, and a “lucky draw” box, students can be more active in the classroom. For example, Child C shared an LtP activity conducted in her class. The teacher asked the children to play a card game in which they had to match two cards to create one complete English word. They handled the cards and moved around the classroom. Through LtP, the children moved around (e.g., from the desk to the floor), made free choices, and engaged in fine motor activities. They also had sufficient opportunities to interact with different people, such as classmates, friends, parents, and siblings. LtP can thus connect multiple learning “partners” through socialized learning activities.

#### 3.2.1. Theme 2: LtP facilitates children's cognitive development

The LtP stakeholders were primarily interested in the extent of students' learning through LtP. The interviewees indicated that children could acquire knowledge, broaden their horizons, improve their memories, and deepen their understanding through LtP. Teacher A explained that children's memories of activities are often more vivid if they enjoy them. As LtP provides multiple learning environments and activities through various devices, children deal with learning content through multiple cognition channels (e.g., visual, auditory, tactile) as they watch, listen, feel, and directly experience. Information processing theory suggests that learning is a complex process involving multiple channels of information that enter the brain and communicate meaning in various ways, including through images, sounds, writing, gestures, facial expressions, and colors. The students scored 4.34 on average for the item “*I understand teachers' instruction more easily in the LtP class*” in the questionnaire.

One child mentioned the “sense of ceremony” in LtP. He believed that quizzes, competitions, props, rewards, badges, and leaderboards create a sense of ceremony in learning. This suggests that feelings can also facilitate children's learning. Teacher B Wang also believed that the elements of maps, badges, and leaderboards teach children visualization techniques that can help them develop their metacognition.

#### 3.2.1. Theme 3: LtP improves various dimensions of an affective learning experience

In the interviews, the students expressed positive attitudes toward LtP and were willing to see more LtP elements in their learning. The nodes for the affective impacts of LtP were most frequently coded in the interview data, compared with its behavioral and cognitive impacts. The affective impacts of LtP were in terms of motivation, relationships, and personality.

First, the children agreed that LtP classes and activities are interesting. LtP stimulates different motivational sources and empowers students in their learning process. Students are motivated to learn for fun or by the desire to compete with others, satisfy their curiosity, achieve goals, or win respect from others, leading to increased engagement in their self-regulated learning.

Second, LtP can help children develop their personalities. As Parent A said, “*we don't want our children to be bookworms. We hope that they are happy and have healthy minds and bodies*.” This opinion was echoed by other parents and teachers. Teacher C explained that China currently pays much attention to children's physical and moral education. As Principal Qian stated, educators' duty is to make children happy and be capable of creating happiness. LtP offers an effective method of achieving this goal. Principal Qian helped teachers develop many LtP projects for the children at his school.

LtP also gives students more opportunities to develop social relationships with teachers, parents, and peers. Child D reported that “*I like teamwork in class. I then know what other classmates do and think*.” In traditional learning activities, students may become tense and bored. Not all LtP experiences are positive. However, children may experience negative effects, mainly in terms of interpersonal problems. More than half of the students reported in the questionnaire that they had experienced verbal conflict (arguments and mocking) and alienation during LtP and 35% of the students reported that they had encountered aggressive behavior.

### 3.3. RQ3: what affects the effectiveness of LtP?

We addressed RQ3 using the questionnaire and interview data to assess internal weaknesses and external influences. First, we assessed the weaknesses and difficulties with LtP using open questions. A total of 113 students reported negative affect experiences when answering the question, “Do you have any problems and difficulties with LtP?” We also interviewed stakeholders to obtain their perspectives on this issue. They reported the difficulties and challenges when designing and implementing an effective LtP. They also discussed the key factors influencing the effectiveness of LtP.

### 3.3.1. Theme 4: internal weaknesses of and difficulties with LtP

Regarding weaknesses, we coded the interviewees' answers in three dimensions: disorder, inefficient teamwork, and limited time and opportunities. First, several LtP activities were deemed disorderly because several naughty children deliberately made trouble. Organizing and managing LtP activities effectively is vital for teachers and parents. Students with limited capacity for self-management found the tasks challenging. Second, LtP teamwork was regarded as inefficient by some of the children and parents. The children typically focus on the devices and tools involved, ignoring the discussion and collaboration components. Finally, several children believed that they had limited time and opportunities to access LtP, as they had extensive homework and tutorial classes.

In addition to the three sub-themes that the children proposed, physical health was an important issue for the parents and teachers. They worried that their children were addicted to playing virtual games. They feared that they would damage their eyesight by staring at the screen for a long time. Several parents were confused about the relationship between exam-oriented education and LtP. Although they wanted their children to be happy, they believed that LtP could not effectively prepare them for exams. Some of the children liked playing educational games on iPads or other smart devices, but their parents did not approve of this approach because they were concerned that excessive screen time would damage their children's eyesight.

### 3.3.2. Theme 5: factors influencing LtP development

From the interviews, the factors influencing LtP development were coded into three categories: subject, environment, and culture. The subjects of LtP mainly include students, parents, teachers, and practitioners (in out-of-school LtP centers). During LtP, children face the challenges of exam stress, social conflict, and game addiction. Effective LtP places relatively high demands on children's self-regulated learning abilities. Three of the four parents identified two main difficulties with LtP. First, they had limited time and energy to participate in their children's LtP activities. Second, they were not fully aware of how to design and evaluate such activities. They often took their children to museums, stadiums, and playgrounds but then let them play alone. The teachers also found it easy to accept the notion of LtP but found it difficult to improve their LtP teaching abilities as they often felt incapable of offering LtP instruction. They became aware of the importance of LtP through various educational reform policies implemented by the government. They believed that more LtP elements could be integrated into their teaching in terms of devices and activities. School campuses could be decorated like amusement parks with many LtP spaces and tools. Teachers would then be required to be gamified learning designers and gaming partners in class. Many LtP pedagogies have been designed that create flexible, interest-driven, and child-centered learning modes.

The four educational officials noted the effects of the environment on LtP development. One educational administrator stated that the rapid development of the economy and technology in China had facilitated the upgrading of spaces and facilities for LtP. Parent B pointed out that, although venue-based learning has recently incorporated several LtP elements, this could still be improved. Venue-based learning could be offered at LtP centers for children. Also, the provision of tailored services for children in living communities is a growing trend in urban areas of China.

Furthermore, digital technologies have led to the creation of numerous LtP tools, but they faced two main challenges. First, parents worry about their children's eyesight and the risk of developing Internet addiction when they spend long periods of time staring at screens. Second, LtP based on digital devices can contribute to the digital gap, in which students with access to devices and resources are better placed to achieve their target learning outcomes.

The most influential factor was culture, which is significant in the education system. Culture can influence learning in terms of concepts, motivations, preferences, and policies. One principal suggested that, in contrast with the traditional view of “playing” as the opposite of learning, parents in China's current cultural setting are currently paying more attention to students' active and positive learning through play, as this makes them happy and engages them. The interviewed parents agreed that LtP has a meaningful role to play in children's learning. They became aware of LtP mainly through the Internet, from teachers, and via recommendations from other parents. The parents of students with low grades welcomed LtP in mainstream courses, but those of high-grade students regarded LtP as a supplement that could be applied when students felt disenchanted with standard learning. In the survey, 40% of the parents felt incapable of guiding their children's LtP regarding design, management, or information literacy. In addition, 36% of them reported that they had little idea about how to choose appropriate LtP tools and products. Due to China's economic progress, educational officials had confidence in the future LtP market. They noted that parents give their children much support in terms of money, time, and attention. In addition, due to the “double reduction” policy (an educational policy in China aimed at reducing children's homework and outside-school tutoring, which was first implemented in 2021), children no longer attend tutorial classes; therefore, the development of the LtP ecosystem is a sign of renewed vitality. This policy was implemented because Chinese children often bear a heavy workload and suffer from stress while learning.

## 4. Discussion

### 4.1. The full picture of LtP development

LtP is an important topic in studies of children's learning, particularly in the current digital and economic climate. This pilot study investigated several issues affecting the development of LtP in China. Other studies (e.g., Turdieva and Olimov, 2021) have focused on educational or serious games as the main forms of LtP or on playful learning. We assessed LtP from a systematic perspective, including the relationships between multimodal learning environments, subjects, objects, activities, and effects. We investigated various LtP spaces, tools, and activities and interviewed participants. According to a review by Lim and Polio (2020), multimodal teaching and learning are based on the organization of semiotic resources (visual, gestural, spatial, linguistic, and others). We found that LtP has gained popularity in both conceptual and practical terms in China. Moreno and Mayer (2007) defined multimodal learning environments as learning environments that use two modes to represent content knowledge: verbal and non-verbal.

**Figure 8:**
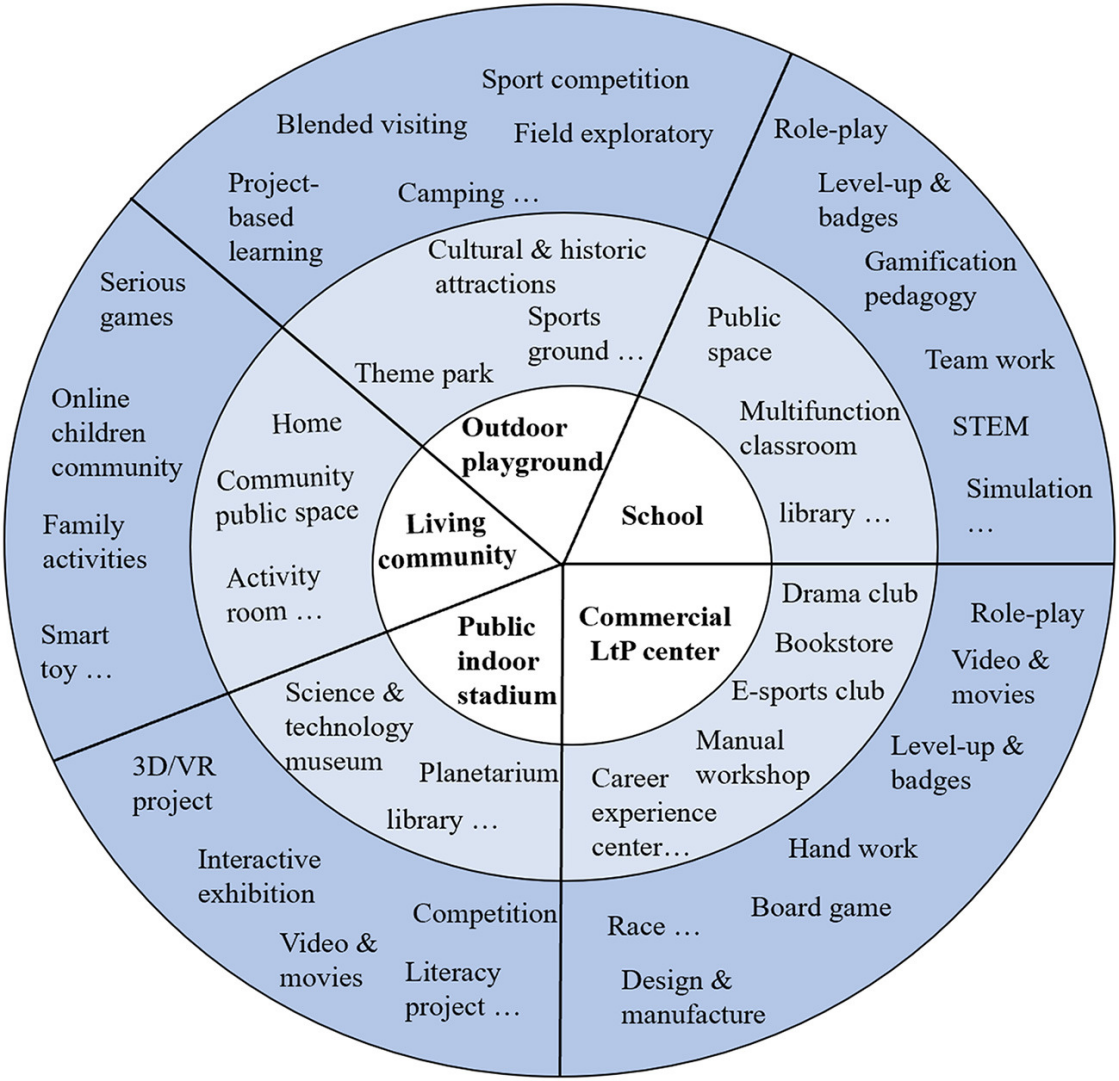
The full picture of LtP spaces and activities.

Our pilot study revealed a more complex structure for the LtP-based multimodal learning environment. As Figure 4 shows, we developed an LtP-based multi-modal learning system that breaks up the monotonous environment of the traditional classroom. As Arnott and Yelland (2021) explained, playful activities can connect children with each other and act as a catalyst for extending visual, aural, spatial, gestural, and linguistic learning modes. The participants' opinions in this study were based on their LtP learning experiences. LtP encompasses various types of formal and informal learning, and LtP projects are typically designed through elements such as game prototypes. These can be fun and interactive, such as role-playing games, action games, virtual manipulation, adventure games, strategy games, battle games, shooting, puzzle games, rewards and leaderboards, card games, sports competitions, music games, social communities, and handicrafts work (Kapp, 2012). Figure 8 shows the broad structure of the LtP environment, in which the inner circle comprises five types of LtP spaces, the second circle comprises LtP places, and the outermost circle comprises LtP activities.

Information technology makes LtP accessible through online and virtual means, resulting in a more diverse and multimodal learning experience. Almost all of the surveyed families reported that their children could access at least one smart device for LtP. Many of the students and parents reported various online or AI-based LtP activities. As digital natives, children in the present times are multiliterate. With the support of technology, the emerging LtP ecosystem has less defined boundaries and more diverse subjects and objects. We found that tablets were the most-used LtP devices. Several researchers have noted a similar phenomenon, suggesting that the iPad has become the most popular portable educational device (e.g., Flewitt et al., 2015). For better or worse, children's skillful use of new technologies, including computers, cameras, iPods, 3D glasses, and smart toys, will continue to expand. They also bring these experiences to school, which affects their ways of learning. The resources and playthings that children use outside of school are increasingly shaping their self-determined learning (Wang et al., 2010; Hirsh-Pasek et al., 2015). As Bustamante et al. (2020) stated, today's digital society offers a multimodal world, and new technologies can be used to interact with the real world. The rapid development of digital technologies enables the construction of rich and diverse learning environments such as online learning, extended reality, and the use of video games. McLean et al. (2017) investigated multimodal learning activities through social media such as Panwapa, Zula World, and Club Penguin, through which children can play games, engage in other activities, and interact with virtual characters. Several LtP centers have begun to make visual diaries for their users, giving children more opportunities to document and create narratives of their playful explorations. If the educational metaverse truly emerges, children will have many more possibilities for LtP.

### 4.2. Multimodal learning effectiveness of LtP

In previous research, the effects of multimodality-based learning were evaluated mainly from the aspect of high-order cognitive abilities and skills. For example, Yelland (2016) proposed that multimodality-based learning helps develop “21^st^ century skills.” Few studies have explored whether there is a form of multimodal learning to improve multiple learning emotions, which are lacking in formal learning. Li and Chu (2021) explained that LtP allows children to engage in playful gaming experiences that enable them to express and communicate their thoughts and feelings using a wide range of learning activities and materials. Therefore, in this study, we investigated the multimodal learning effectiveness of LtP from three aspects: behavioral, cognitive, and affective. First, LtP can improve multiple learning behaviors compared to other forms of multimodal learning as it involves various gaming behaviors and actions (Turdieva and Olimov, 2021). Games often perform many behavioral modes at once: view, listen, speak, write, communicate, move, etc. Through LtP, children can operate tools, handle props, express their emotions, move freely, and have more chances for practice in various environments. According to embodied cognition theory (Shapiro, 2010), motor and sensory systems are fundamentally integrated into cognitive processing.

Second, this study found that LtP can promote students' cognitive and meta-cognitive competence. Consistent with other studies (e.g., Blikstein and Worsley, 2016), this study shows that the setting of multimodal assignments in LtP makes learners understand knowledge easier, according to students' reports. Cognitive learning theory (Greeno et al., 1996) suggests that, when learners are actively involved in their own learning, they retain more of their training; therefore, multimodal assignments are likely to lead to better learning results (Lim and Polio, 2020). The phrase “sense of ceremony,” as expressed by one student interviewee, can explain how sensory input is integrated with the learning process. “Sense of ceremony” is a buzzword in contemporary Chinese society. It emphasizes the importance of taking life seriously and expressing one's emotions with formality and dignity (Mi et al., 2021). The student felt that his teachers' gamified pedagogy helped to develop a sense of ceremony through points awarded, leaderboards, “blind boxes,” and an “answer bell.” Xiong (2021) also emphasized the importance of a sense of ceremony in online classes, to which LtP can be added through various game props.

Furthermore, it has been found that LtP can develop children's meta-cognitive competence, such as self-regulated learning abilities. Children generally find it hard to keep still and listen to teachers for long periods, while LtP allows children to learn to control themselves according to gaming rules. During LtP activities, they learn to manage time, use strategies, and utilize tools and technologies.

The clearest effectiveness offered by LtP is its positive influence on students' multiple emotions and level of motivation, which is also the main reason for its popularity. Playing and gaming have been found to increase students' interest in learning and motivation to learn. To solve a problem and complete a playful task, children try their best to remember, understand, and apply knowledge (Liu et al., 2017). Social affect development is another meaningful result of LtP and represents a particular contribution of LtP to multimodal learning. Although children may encounter interpersonal problems during LtP, they learn how to face and solve them (Mørch et al., 2015). The scenes are real and cannot be experienced in traditional classes. In addition, LtP can improve family relationships. When children complete their homework with their parents' support, a tangible link is established between home and school. LtP is a harmonious way to create such a link, allowing children to learn more effectively and parents to communicate more effectively with their children. Our study was conducted during the COVID-19 pandemic, a time when staying at home gave children and parents more chances to engage in LtP. This may be one reason the parents appeared familiar with LtP.

### 4.3. How to improve the effectiveness of LtP

This study revealed three main factors influencing LtP: subjects, environment, and culture. The subjects involved in LtP are students, teachers, parents, and practitioners. Their views, attitudes, and abilities affect their acceptance of LtP to a large extent. Parents' educational backgrounds and family SES also affect the practice of LtP. Environmental factors include spaces and tools, which can be online or offline. We found that most of the sampled children and parents preferred offline LtP spaces but still liked using smart devices. As Kumpulainen et al. (2020) stated, digitization has a significant effect on children's entertainment and learning. Culture plays an important role in the development of LtP worldwide, as it is shaped through traditions and customs, language, religion, social rules, economic structures, and governance policies. These can determine the level of a group's acceptance of LtP. In traditional Chinese culture, effective learning is regarded as the “unity of knowledge and action.” Through experiences with various places and people, a multimodal learning experience can be obtained. The rich content of Chinese culture strengthens the connection between LtP and multimodal learning. With the development of the Chinese economy, families' financial investment in education has increased dramatically. In this study, most of the urban Chinese children sampled had access to at least one type of smart device in addition to smartphones. Children in developed areas of China have ample LtP resources and support from their families, schools, and society. Bankler (2019) discussed the cultural adaptation of playful learning in China, noting that China is the world's largest market for educational games. Dong and Mangiron (2018) suggested that several cultural elements should be considered when designing educational games for China, such as songs, colors, myths and legends, props, and traditional toys.

Based on our survey results, we offered several practical suggestions and strategies for developing LtP. First, the environment of tools and resources for LtP should be further improved. More well-designed LtP spaces should be added to campuses and other sites, and they should also offer students a multisensory experience. Traditional Chinese educational toys could be adapted to be AI-based learning products. Second, the relevant abilities and attitudes of stakeholders should be developed. Teachers and parents should be both mentors and participants in students' LtP activities, both inside and outside of school. Parents and teachers require more support and guidance to design and implement LtP. Third, stakeholders should reform curricula and educational evaluations related to LtP. The interviewed teachers also reported that they needed clearer information regarding the links between LtP and the national curriculum standards, ideally through official educational policies. Out-of-school LtP organizations and products should be classified, evaluated, and supervised. LtP has become a new business stream in the after-school child market, and numerous educational and technology enterprises have created innovative LtP centers for children and families. Policymakers hope that the LtP industry will flourish and be competitive. The development of an LtP culture requires the joint efforts of families, schools, and society.

## 5. Limitations and conclusion

In this study, a large-scale survey was conducted in developed cities in China to assess the current status of LtP. We offered an overview and constructed a practical ecosystem for multimodal learning. However, several limitations should be recognized. First, our study focused on samples and cases from four first-tier cities in China, representing China's highest economic and educational levels. There may be a major divide in LtP—both in theory and in practice—between such cities and rural areas. Second, we focused on the LtP-based multimodal learning ecosystem rather than explaining the multimodal learning mechanism of LtP. Our multimodal learning analysis of LtP was limited. The association between LtP and multimodal learning may therefore appear weak.

Further empirical research could illustrate the direct relationship between LtP and multimodal learning. Finally, this study focused on physical and online LtP spaces and activities rather than completely virtual LtP. As the educational metaverse emerges, future LtP studies should investigate online LtP in more detail.

This study examined the multimodal learning experience of LtP in Chinese urban areas. The affordances of LtP in such learning experiences arise from its multimodal environments, subjects, events, and results. Despite the advantages of deep multimodal learning, several difficulties and challenges must be overcome before an open, effective, and fun LtP ecosystem can be developed. In summary, the notion of LtP and its implementation can enrich the development of multimodal approaches, leading to deep and meaningful learning. The LtP ecosystem is a creative learning ecology that can result in high-quality educational innovation. As we continue to steer our children's development, they will undoubtedly require a wider range of enriching learning opportunities in terms of places, forms, and people. LtP has great potential to achieve this goal by encouraging children's natural curiosity, engagement, social connection, and independent thought.

## Data availability statement

The original contributions presented in the study are included in the article/Supplementary material, further inquiries can be directed to the corresponding author.

## Ethics statement

The studies involving human participants were reviewed and approved by Peking University. Written informed consent to participate in this study was provided by the participants' legal guardian/next of kin.

## Author contributions

XL, XZ, YZ, LZ, and JS: conceptualization and writing—review and editing. XL, YZ, and JS: methodology. YZ, LZ, and JS: investigation. XL, XZ, and YZ: data analysis with constructive discussions and writing—original draft preparation. XL, LZ, and JS: project administration and resources. XL and JS: funding acquisition. All authors have read and agreed to the published version of the manuscript.
