# Supplementary material for: Exploring the role of learning through play in promoting multimodal learning among children: a pilot study in Chinese first-tier cities
Source: Front Psychol. 2023 May 30;14:1103311. doi: 10.3389/fpsyg.2023.1103311 (PMC10263124; doi:10.3389/fpsyg.2023.1103311)
Supplement: Supplementary file 1 [file Data_Sheet_1.pdf]

Appendix 1: LtP Survey Scale

Notes: the introduction and demographic characteristic questions are deleted here.

1. Your acceptance of LtP (five-point Likert Scale)

**Dimension**	**Items**
Use accessibility	I have a lot of time for LtP<br>I think LtP become more both in and out class.<br>My parents support me for LtP<br>My teachers support LtP and often use LtP in class<br>LtP enables me to try various possibility of learning
Usefulness recognition	Learning Through Play make my learning joyful.<br>Learning Through Play make my learning meaningful.<br>Learning Through Play make me engage in learning more actively.<br>Learning Through Play allow me to try and practice repeatedly.<br>Learning Through Play give me chance to interact with others.
Use Intention	I like to learn through LtP in class.<br>I will try to introduce LtP to my teacher.<br>I will try to introduce LtP to my schoolmates.<br>I am willing to do a job about Learning Through Play.<br>I think LtP is not fun as entertainment games.

2. To what extent you like the following LtP spaces (five-point Likert Scale) Outdoor theme playground

- Commercial play center
- public science and technology museums
- School space
- Living community space

3. From the following tools that can be used for LtP, choose one that you most-often use and the one you favorite

- Smartphone
- Tablet
- Learning Robot
- Game console (e.g., handle game)
- Smart glasses (e.g., VR/CR games)

4. To what extent you like the following LtP activities (five-point Likert Scale)

- Watch video and experience VR
- Participate in LtP group projects
- Read and write
- Watch the exhibition

5. To what extent you like the following future LtP characteristics (five-point Likert Scale)

- Hands-on activities

- High-tech devices and tools
- Group LtP activities
- Together with parents
- Open and outdoor LtP space
- Colorful and comfortable spaces
- With tutors or instructors

6. To what extent you agree the effects of LtP (five-point Likert Scale)

**Dimension**	**Items**
Behavior-4	Learning Through Play make me engage in learning more actively.<br>Learning Through Play allow me to try and practice repeatedly.<br>Learning Through Play give me chance to interact with others.<br>Learning Through Play can accelerate my learning speed.
Cognition-4	• LtP can increase my extra-curricular knowledge<br>• I become more positive and active when thinking in LtP classes<br>• I think I am more able to find out that my understanding of some instruction content was wrong, and needed to be corrected in LtP classes.<br>• I understand the instruction content better in LtP classes
Affection-4	• Learning Through Play make my learning joyful.<br>• Learning Through Play make my learning meaningful.<br>• I think the relationship between I and my classmates become friendlier in LtP<br>• I feel more confident and braver in LtP

Open question:

- please recommend your favorite one and explain the corresponding reasons
- Please report your difficulties and challenges for effective LtP
- Please provide suggestions for the development of effective LtP

Appendix 2: The outline of the semi-structured interviews

Participants	Interview outline
Children	• What LtP experience did you have<br>• What can you do in LtP<br>• What do you receive from LtP<br>• What is your expectation of LtP in the future<br>• What are challenges or barriers of LtP from your opinion
Teachers	• How do you implement LtP in the classroom<br>• What are the effects of LtP from your opinion<br>• What are challenges or barriers of LtP from your opinion
Parents	• What are the effects of LtP from your opinion<br>• How do you participate in children's LtP<br>• What challenges or barriers of LtP from your opinion What are your expectation of LtP in the future
Educational administrators	• What are the roles of LtP in the educational system from your opinion<br>• What are effects of LtP from your opinion

	• What are challenges or barriers of LtP from your opinion<br>• How do we implement effective LtP from your opinion
